# International consensus recommendations on face transplantation: A 2-step Delphi study

**DOI:** 10.1016/j.ajt.2023.08.023

**Published:** 2024-01

**Authors:** Benedetto Longo, Fay Bound Alberti, Bohdan Pomahac, Julian Joseph Pribaz, Jean-Paul Meningaud, Benoît Lengelé, Ömer Özkan, Özlenen Özkan, Juan Pere Barret, Patrik Lassus, Phillip Blondeel, Nathalie Roche, Raffi Gurunian, Pedro Infante-Cossio, Andrew Lindford, Gerald Brandacher, Pietro Giovanoli, Jan Plock, Vijay S. Gorantla, Emily Ruppel Herrington, Daniel Saleh, Ibrahim Natalwala, Massimo Cardillo, Sheila Jowsey-Gregoire, Simone La Padula, Derek Manas, James Benedict, Gloria Nuccitelli, Romain Bosc, Roberto Morello, Anneke Farías-Yapur, Martina Giacalone, Sarah Hall, Gennaro D’Orsi, Valerio Cervelli

**Affiliations:** 1 Chair of Plastic Surgery, Department of Surgical Sciences, Tor Vergata University of Rome, Rome, Italy; 2 Director of Interface and Director of the Centre for Technology and the Body, King’s College London; 3 Division of Plastic and Reconstructive Surgery, Department of Surgery, Yale New Haven Hospital, Yale School of Medicine, New Haven, Connecticut, USA; 4 Department of Plastic and Reconstructive Surgery, Morsani College of Medicine, University of South Florida, Tampa, Florida, USA; 5 Department of Plastic, Reconstructive, and Maxillofacial Surgery, Henri Mondor Hospital, University of Paris, Créteil, France; 6 Department of Plastic and Reconstructive Surgery, Cliniques Universitaires Saint-Luc, Brussels, Belgium; 7 Department of Plastic and Reconstructive Surgery, Akdeniz University School of Medicine, Antalya, Turkey; 8 Department of Plastic Surgery and Burns, Vall d’Hebron Barcelona Hospital Campus, Universidad Autònoma de Barcelona, Barcelona, Spain; 9 Department of Plastic Surgery, Helsinki University Hospital, University of Helsinki, Helsinki, Finland; 10 Department of Plastic and Reconstructive Surgery, Ghent University Hospital, Ghent, Belgium; 11 Department of Plastic Surgery, Cleveland Clinic Abu Dhabi, Abu Dhabi, United Arab Emirates; 12 Department of Oral and Maxillofacial Surgery, Virgen del Rocio University Hospital, University of Seville, Seville, Spain; 13 Department of Plastic and Reconstructive Surgery, Johns Hopkins University School of Medicine, Baltimore, Maryland, USA; 14 Department of Plastic Surgery and Hand Surgery, University Hospital Zurich, Zurich, Switzerland; 15 Department of Surgery, Wake Forest School of Medicine, Wake Forest Institute of Regenerative Medicine, Winston Salem, North Carolina, USA; 16 Department of Communication, University of Pittsburgh, Pittsburgh, Pennsylvania, USA; 17 Department of Plastic and Reconstructive Surgery, The Newcastle upon Tyne Hospitals NHS Foundation Trust, Newcastle upon Tyne, UK; 18 Department of Plastic Surgery, Leeds Teaching Hospitals, Leeds, UK; 19 Director of National Transplants Center, National Institute of Health, Italian Ministry of Health, Rome, Italy; 20 Department of Psychiatry and Psychology, Mayo Clinic, Rochester, Minnesota, USA; 21 Department of Plastic and Reconstructive Surgery, Università degli Studi di Napoli Federico II, Napoli, Italy; 22 NHS Blood and Transplant, Stoke Gifford, Bristol, UK; 23 Liver Transplant Unit, Freeman Hospital, Newcastle Hospitals NHS Foundation Trust, Newcastle University, Newcastle, UK; 24 Center for Global Health Ethics, Duquesne University, Pittsburgh, Pennsylvania, USA; 25 Division of Anesthesia and Intensive Care Medicine, Department of Clinical and Surgical Translational Medicine, Sant’Andrea Hospital, Sapienza University of Rome, Rome, Italy; 26 Department of Maxillofacial Surgery, Sant’Andrea Hospital, Sapienza University of Rome, Rome, Italy; 27 School of Psychology, Universidad Panamericana, Benito Juárez, Mexico City, Mexico; 28 AboutFace, University of York, UK

**Keywords:** face transplantation, consensus recommendations, vascularized composite allotransplantation

## Abstract

Face transplantation is a viable reconstructive approach for severe craniofacial defects. Despite the evolution witnessed in the field, ethical aspects, clinical and psychosocial implications, public perception, and economic sustainability remain the subject of debate and unanswered questions. Furthermore, poor data reporting and sharing, the absence of standardized metrics for outcome evaluation, and the lack of consensus definitions of success and failure have hampered the development of a “transplantation culture” on a global scale. We completed a 2-round online modified Delphi process with 35 international face transplant stakeholders, including surgeons, clinicians, psychologists, psychiatrists, ethicists, policymakers, and researchers, with a representation of 10 of the 19 face transplant teams that had already performed the procedure and 73% of face transplants. Themes addressed included patient assessment and selection, indications, social support networks, clinical framework, surgical considerations, data on patient progress and outcomes, definitions of success and failure, public image and perception, and financial sustainability. The presented recommendations are the product of a shared commitment of face transplant teams to foster the development of face transplantation and are aimed at providing a gold standard of practice and policy.

## Introduction

Prospective research on face transplantation (FT) dates to early 1991,^1^ although it was not until 2005 that the first procedure was performed.^2^ To date, FT is recognized as a viable procedure to treat severe facial defects not amenable to conventional reconstruction, or which have been treated with standard reconstructive procedures, albeit with suboptimal aesthetic and functional outcomes. Over the last 17 years, 48 procedures have been performed worldwide.^3^ FT is a complex procedure bearing significant risks of complications and mortality, requiring life-long care, and posing major psychosocial challenges.^4^ The paucity of cases, the heterogeneity of candidates and contexts, and communication gaps among teams have hampered the development of standardized processes for candidate selection, indications and outcome evaluation, and reporting. Furthermore, lack of updated literature on long-term outcomes and quality of life and the absence of agreed protocols for outcome evaluation impeded to reach consensus on success and failure. Follow-up of FT recipients has shown allograft vulnerability to chronic immunologic rejection, urging attention from FT teams with regard to salvage strategies. Despite the challenges experienced, media reports of outstanding results fail to depict FT in its wholeness. FT is a life-enhancing procedure requiring thorough evaluation and risk-benefit assessment. In virtue of this, all patients and their social support networks should be informed about the risks the procedure poses, and the economic burden it implies, and be prepared to engage in a life-long path. The cost of FT and life-long care, coupled with the lack of financial assistance by government institutions or private insurance, significantly impacts the economic viability of FT, introducing a structural bias of access to the procedure. In the light of these issues, the Policy Institute at King’s College London, with the AboutFace project, based at the University of York, convened a group of international experts in FT to discuss many of the questions remained unanswered.^5^ The collaboration of major stakeholders in the field brought to the definition of a set of consensus recommendations, which serve as a gold standard of practice and care.

## Methods

The study comprised 5 stages: (1) literature search, (2) prepolicy laboratory survey, (3) AboutFace policy laboratory, (4) postlaboratory survey, and (5) analysis of survey responses, discussion, and drafting of final recommendations (Fig). The consensus method was based on the modified Delphi methodology following the RAND/UCLA recommendations. The Delphi method is characterized by 4 key aspects: (1) a group of experts is surveyed regarding the topic of interest; (2) anonymity is maintained throughout the process to prevent conformity to dominant views; (3) the approach is iterative, involving multiple rounds; and (4) the design of subsequent rounds is dependent on the responses gathered from the previous round. The adoption of Delphi methodology enables to build structured anonymous communication among individuals sharing expertise in a specific subject, with the goal of reaching a consensus within a broader group.

**Figure:**
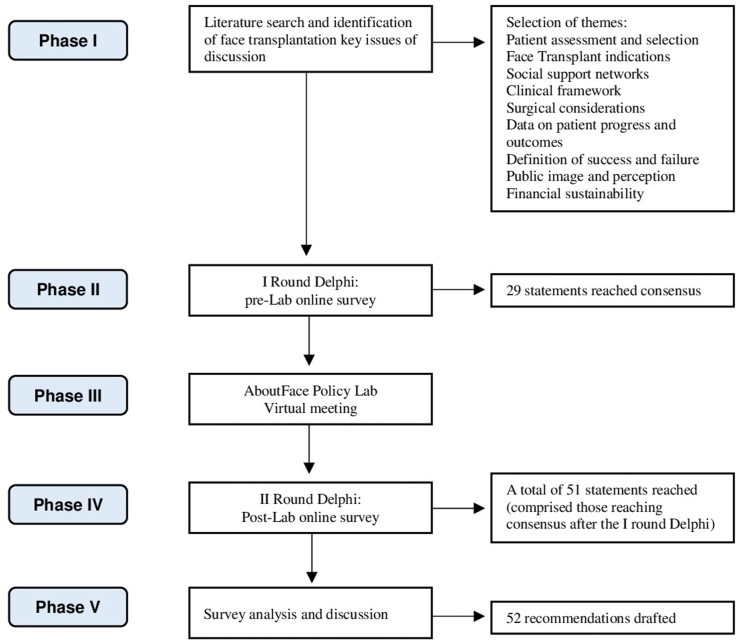
Study design: The 2-step Delphi process.

### The AboutFace project

AboutFace was an interdisciplinary research initiative headquartered at the University of York led by Prof. Fay Bound Alberti and supported by a UKRI Future Leaders Fellowship. In 2023, the project moved to King’s College London, where it has been rebranded as Interface. Its primary focus revolves around studying the historical aspects of FTs and delving into their psychosocial and cultural significance. It is based on the collaboration with surgical teams, patients and families, organizations, and policymakers to assess the effects of FTs on patients, practitioners, donors, and families. Additionally, it aims to investigate the influence of media representations on policy and public opinion. AboutFace also evaluates FT as an innovative surgical technique while exploring the interconnections between ethics, emotions, identity, and facial appearance.

### Panel formation and 2-step Delphi process

A steering committee at the AboutFace research project selected and invited a panel of experts based on the following criteria: (1) those who are medical specialists with clinical or research expertise in FT (one or more representatives of all FT teams were invited); (2) those having a publication track record in the field of FT; and (3) those who are nonmedical specialists, with substantial knowledge on FT.

In December 2021, AboutFace, at the University of York and the Policy Institute at King’s College London, organized a 3-day online policy laboratory to discuss and build a collaborative blueprint for sustainable FTs. A 2-step Delphi method was adopted to develop the consensus recommendations among the panel. Before the virtual meeting, AboutFace selected issues of discussion based on in-depth quantitative and qualitative research undertaken over 2 years. Nine themes were selected and addressed in dedicated sections (Fig). Invited delegates were asked to complete a prelaboratory survey, the first Delphi questionnaire, to capture views and define areas of consensus and disagreement. During the laboratory test, participants were split into interdisciplinary breakout groups to discuss key issues pertaining to the selected themes and analyze survey replies. Comments and suggestions received after the prelaboratory survey and the policy laboratory online meeting were incorporated into the second round of Delphi, for which a 78-question survey was designed (Supplementary Methods). When necessary, questions were revised and reiterated, and new questions were introduced to provide more granularity and detailed statements in the second Delphi round. Survey answers were recorded using a 5-point Likert scale: (1) strongly agree, (2) somewhat agree, (3) neutral, (4) somewhat disagree, and (5) strongly disagree. A free-text section for suggestions and comments was also included. An access link to the online survey was provided via email. Participants were asked to complete the survey and confirm their willingness to contribute to the final steps of the project, entailing the analysis of 2-step Delphi questionnaires and the drafting of recommendations. The survey was open for a period of 6 weeks. Two reminder emails were sent before the deadline. Survey replies remained anonymous.

### Consensus establishment

The 2-round Delphi survey results were analyzed using the standardized criteria for agreement based on the RAND method.^6^ For the statements for which a response was recorded using the Likert scale, the categories strongly agree and somewhat agree, or strongly disagree and somewhat disagree, were considered together. Final recommendations were drafted as follows: ≥75% agreement was classified as a strong agreement for a statement, whereas ≥75% disagreement indicated a strong disagreement. Statements failing to achieve the consensus criteria even after the second round of Delphi were recorded as areas of dissensus.

## Results

Thirty-five among 51 invited experts completed the 2-round Delphi process, between November 2021 and November 2022. The panel group included members of 10 FT teams, with a representation of 35 out of the 48 FTs performed (73%). The panel included 25 surgeons and clinicians (71%), and 10 nonmedical experts (29%) (psychologists, qualitative researchers, policymakers, and members of advocacy groups). Consensus was reached on 29 statements after the first Delphi round, and on a total of 52 statements after completion of the second round. Statements reaching consensus were shared with and approved by the panel before the drafting of recommendations. A total of 52 recommendations were produced (Table).

**Table:** Consensus recommendations on face transplantation.

**Patient assessment and selection** 1. The development of standardized protocols for face transplantation candidate assessment and selection is required. 2. Patient assessment and selection should be handled by multidisciplinary teams of experts (surgeons, clinicians, psychologists, and psychiatrists), with the aim of evaluating candidate clinical and psychosocial suitability to face transplantation. 3. Besides clinical conditions, individual circumstances, including psychological status, personal distress, and self-perception, must be thoroughly assessed and considered as part of the patient assessment process. 4. Patient expectations and perception of “transplant success” should be investigated and discussed at the time of patient enrollment in a face transplant program. 5. Preoperative psychological and psychiatric evaluations are mandatory for enrollment in a face transplant program. 6. The presence of a well-established social support network is a basic requirement for face transplantation. Accordingly, in the absence of a social support network, the indication to face transplantation should be questioned.
**Indications** 1. Face transplantation is indicated for the treatment of extended craniofacial defects and defects involving mid and central aesthetic facial units. 2. Face transplantation might be considered as a first-line approach, following a thorough benefit vs risk evaluation, for craniofacial defects involving key anatomical and functional structures and/or so extensive to predict a suboptimal outcome of conventional reconstruction. 3. Face transplantation is indicated for defects with a complete loss of the orbicularis oculi and/or orbicularis oris muscles. 4. A past medical history of benign tumor should not be considered as a contraindication to face transplantation.
**Social support networks** 1. When assessing patient social support networks, psychosocial support (a positive attitude to promote patient well-being, self-acceptance, social reintegration, and return to work) should be investigated. 2. When assessing patient social support networks, practical support (availability to assist the patient on follow-up course, rehabilitation, revision surgeries, return to work, and social reintegration) should be investigated. 3. When assessing patient social support networks, financial resources (financial capacity to ensure life-long immunosuppressive therapy, psychological/psychiatric support, rehabilitation, follow-up-related costs, travel, and housing costs) should be investigated. 4. Social support network members should be involved as soon as possible in the face transplant process. 5. Face transplant teams must ensure open discussions on quality of life after transplant, transplant-related complications, allograft loss, and chronic immunologic rejection with social support network members.
**Clinical framework** 1. Collaboration and sharing of information among clinical teams, on a national and international basis, is necessary to improve single-team capacity and ensure the best care to face transplant recipients. 2. A comprehensive posttransplant follow-up should provide clinical and psychological care to face transplant recipients, handed over multidisciplinary teams of experts. 3. Investigation of predictive factors of success (trauma-related distress, psychological assessment, social outcomes, and biomarkers) is required. 4. Investigation and research on immunosuppressive therapy protocols are necessary to minimize the risk of immunosuppression-related complications (eg, infections and tumors), to which all transplant recipients are inevitably exposed and because of which a shorter than normal life expectancy is expected. 5. The development of standardized processes for monitoring the immunologic rejection of face allografts (follow-up mucosal/skin biopsies, study of vascular changes through echo/magnetic resonance imaging, and serum antibody titers) is necessary. 6. Chronic immunologic rejection causing late allograft loss is a major challenge in the long-term follow-up of face transplant patients. 7. The risk of chronic immunologic rejection should be discussed with face transplant recipients, outlining the possible need for future reconstructions with autologous tissues or a new face transplantation. 8. A comprehensive face transplant plan must address salvage contingency strategies to adopt in the unlikely event of face allograft failure/loss. 9. Facial retransplantation is a valid approach in case of face allograft loss.
**Surgical considerations** 1. In the setting of donor surgery, performing face allograft procurement at first, and under good hemodynamic donor conditions, is desirable. 2. In case of donor hemodynamic instability, organ procurement must have the priority and face allograft harvest should be aborted or postponed. 3. Face donor should be free of any kind of probes (ie, venous access and nasogastric tube) to avoid any interference with allograft procurement. 4. Tracheostomy is required to perform face allograft procurement. 5. Allograft ischemia time should be minimized; ideally a cutoff of <4 h is advised. 6. A respectful return of the donor to the family should be granted, according to the family’s wishes (eg, facial mask). 7. Recipient surgery should be started once face allograft procurement has been done or has been considered safely performed by the donor team.
**Data on patient progress and outcomes** 1. Formalized processes, ideally a data registry for all face transplant procedures, are required to ensure timely reporting and sharing of posttransplant outcomes (eg, rejection episodes, posttransplant complications, motor and sensory recovery, and posttransplant quality of life) among face transplant teams, on a national and international basis. 2. Data collection and sharing is necessary to improve patient and social support network evaluation. 3. Outcome reporting and analysis are necessary to promote the development of funding models for face transplantation. 4. Standardized metrics are required to evaluate posttransplant individual outcomes (quality of life, self-perception, social reintegration, and return to work). 5. Electronic scheduled collection of questionnaires on patient’s perception and experience following transplantation (PROMS, PREMS, and QoLs) should be considered as a measure to improve completion rates. 6. The quality of life and outcomes of patients receiving face transplantation should be compared with that of those referred for face transplantation, who decide not to proceed with the surgery, with the aim of understanding potential benefits and the impact of face transplantation on patients with severe craniofacial defects.
**Definitions of failure and success** 1. A consensus definition of failure in the setting of face transplantation is necessary. 2. Reaching a consensus definition of failure in the setting of face transplantation is required to improve patient selection and foster investigation on pathophysiological mechanisms of failure-related causes. 3. A comprehensive definition of failure in the setting of face transplantation must include all conditions leading to irreversible deterioration of the face allograft (eg, surgical complications, immunologic rejection, and allograft vasculopathy). 4. A comprehensive definition of failure in the setting of face transplantation must include all external conditions prompting removal of the face allograft (eg, infections, cancer, and severe systemic complications). 5. A comprehensive definition of failure in the setting of face transplantation must include “preventable” externalities related to patients’ compliance and posttransplant experience (eg, suicide, noncompliance to immunosuppressive therapy, and follow-up). 6. A comprehensive definition of failure in the setting of face transplantation must include patient death for transplant and immunosuppressive therapy–related complications (eg, tumor occurrence and opportunistic infections). 7. It would be advisable to distinguish failures related to conditions directly affecting the viability of face allografts from deaths related to suicide, noncompliance, and immunosuppression-related complications. 8. Being a life-enhancing procedure, success in the setting of face transplantation must be evaluated beyond clinical and surgical aspects, considering patient’s posttransplant self-perception, experience, and quality-of-life change. 9. Success in the setting of face transplantation is strongly affected by patient and social support network pretransplant expectations on outcomes and posttransplant course. Accordingly, any effort should be made to depict to face transplant candidates and their support networks’ practical aspects of life after transplantation (rehabilitation, need of revision surgeries, psychological support, immunosuppressive therapy, and follow-up visits).
**Public image and perception** 1. Comprehensive, long-term, patient-centered narratives, including individual experience, the role of social support networks, and challenges faced after face transplantation, should be encouraged over brief reports and before-and-after comparisons. 2. Face transplant programs should provide patients with proper instruments to deal with media interest and to avoid violation of their privacy.
**Financial sustainability** 1. Funding systems are necessary to ensure face transplant economic sustainability for patients and to avoid the structural bias of face transplantation access. 2. Data on posttransplant outcomes and QoL measures may be an instrument to depict the benefits of face transplantation as opposed to other treatments or nontreatment to funders. 3. Standardization of patient assessment and selection may provide an additional tool to encourage the funding of face transplantation. 4. Institutions should provide the necessary long-term financial support to face transplant recipients as a gold standard of care. This must include costs of housing, travel, follow-up, biopsies, and immunosuppressive therapy.

PREMS, patient-reported experience measures; PROMS, patient-reported outcome measures; QoL, quality of life.

## Discussion

### Patient assessment and selection

Assessment and selection of FT candidates is a key challenge for FT policy and practice. FT teams at different institutions have proposed valuable tools, although a shared set of criteria has not been established yet.^7^^,^^8^ Lack of standardization in patient selection is partly due to the small number of patients involved and also a result of lack of standardized measures for outcome assessment and inadequate outcome data sets. FT stakeholders agreed on the need to develop standardized protocols for evaluation of candidate suitability for FT. According to the panel, a thorough evaluation of FT candidates should be handled by multidisciplinary teams. Besides the assessment of the clinical status, the individual dimension should be investigated, including patient psychological status, distress, and self-perception. Although these conditions might be difficult to assess, psychological and social resilience has shown to be crucial for transplant success, given the challenges of rehabilitation, social reintegration, and immunosuppression, with which FT recipients must cope life-long.^9^ Psychological and psychiatric evaluation was deemed mandatory prior to transplantation, whereas conflicting viewpoints were noted on whether a history of psychiatric illness might be a contraindication (47% agreement, 32% disagreement, and 20% neutral). The evaluation of patients with a history of psychiatric illness, self-harm, and addictive behavior is certainly challenging. However, histories of success among these patients have been reported, with FT being a second chance at life. Case-by-case evaluation appears to be the most reasonable choice. Participants recommended investigation of patient expectation and individual perception of transplant success to promote open discussion with the clinical team prior to transplantation. Furthermore, assessment of the candidate’s social support network was recognized as a key part of a comprehensive patient evaluation, in the absence of which candidate suitability should be questioned.

### Face transplant indications

From 2005, 48 FTs were performed in patients with severe facial defects resulting from trauma, burns, neurofibromatosis, animal attacks, chronic rejection of face allograft, tumors, and arteriovenous malformation.^10^ FT centers have shared unique inclusion/exclusion criteria for FT, although commonly shared prerequisites have not been established yet. The heterogeneity among candidates, types of defects, and extended patient clinical and psychosocial frameworks require case-by-case evaluations and risk-to-benefit analysis.^11^ Despite the need of tailoring the indication, some key shared recommendations were drafted from expert opinions. Consensus was reached on the indication to FT for extensive facial defects and defects of the midface, involving key functional anatomical structures (eg, orbicularis oris and orbicularis oculi muscles), regardless of defect-related circumstances. Most FT recipients access FT programs after having undergone multiple reconstructive procedures. This has raised the question of whether FT might be considered as a first-line approach, rather than as a last resort. Only one case of FT as a first-stage procedure has been reported in literature to date.^12^ According to participants, FT should be considered as a first-line approach for those defects that are so extensive or involve key structures, for which a suboptimal outcome of conventional reconstruction is predictable. FT stakeholders were interviewed about clinical conditions that might represent contraindications to FT. A history of benign tumors was not deemed a contraindication, whereas no consensus was reached on the indication to FT in patients with a history of malignancy, even after a 20-year free disease period. As regards HIV infection, which counts a single case among FT recipients, contradicting views were noted, and no consensus was reached.^13^

The evaluation of children as potential FT candidates has been commented on by leaders in vascularized composite allotransplantation, psychologists and psychiatrists, ethicists, and researchers. Barriers precluding inclusion of pediatric patients among potential candidates include the difficulty of establishing whether and at which age a child might be aware of the procedure implications, parents’ right to give consent, identity and psychological issues faced with growth, long-term denial, and the plausible trade-off for a better, but shorter, life.^14^, ^15^, ^16^ Agreement was not reached, although 40% of delegates were in favor of extending the indication to pediatric patients. A further issue of discussion is the clinical feasibility and ethical aspects of performing cross-sex FT. Although no consensus was reached, 48% of the panel deemed donor and recipient sex matching not strictly necessary.

### Social support networks

As discussed in section “Patient assessment and selection,” support networks are crucial to determine whether a patient might be a good fit for an FT. According to the panel, a negative attitude of the care providers toward FT might severely affect recipient acceptance of the face allograft, thereby increasing patient psychological distress, anxiety, and threatening compliance to follow-up. Social support networks should be assessed on multiple fronts, including psychosocial attitude, practical aid, and economic resources. Survey participants also recommended involvement of support network members from the early phases of FT. Open discussions with the clinical team are necessary to prepare both patients and support network members. These should raise awareness on posttransplant realities and understand whether adequate support may be possible. The panel recommended transparent discussion on transplant-related challenges, including complications, rejection, and allograft loss, and the possible need to resort to conventional reconstruction or retransplantation. Assessing whether patients or social support networks have adequate economic resources to ensure life-long care is fundamental, albeit it creates a structural bias related to socio-economic conditions. Survey participants agreed on the importance of promoting economic sustainability of FT, through alternative funding systems, which might mitigate such health inequality. One more suggestion was to foster communication and sharing of experiences between FT recipients and social networks with candidates and their families.

### Clinical framework

FT is a life-long process entailing multiple clinical contacts and posing challenges to both patients and FT teams, from pretransplant assessment to long-term follow-up care. The commonest complications witnessed among FT recipients include infections, de novo metabolic alterations, and tumor occurrence.^17^As regards immunologic tolerance, acute rejection is rather common in the posttransplant course, whereas chronic rejection has emerged as a new threat to long-term allograft survival.^18^^,^^19^ Laboratory discussion and survey analysis yielded consensus from the experts on general measures for practice and suggestions on future research directions. Participants agreed on fostering collaboration and data sharing among FT teams, on an international basis, to improve single-team capacity and ensure the best care to FT recipients. As regard to long-term care, the heterogeneity of contexts makes the development of standardized programs somewhat difficult. According to the expert group, any FT program should ensure a posttransplant follow-up, handled by multidisciplinary teams, which includes long-term clinical and psychological care. FT stakeholders recognized the urgent need to address the issue of chronic immunologic rejection and allograft loss. Agreement was found on the development of standardized processes for monitoring rejection of face allografts (follow-up biopsies, echo/magnetic resonance imaging, and serum antibody titers). Furthermore, participants recommended the implementation of FT plans with contingency strategies and salvage options to undertake in the event of allograft failure.^20^^,^^21^ Participants outlined the importance of patient and support network awareness, recommending discussion with the clinical team on chronic rejection and its implications, prior to transplantation. Furthermore, the importance of implementing research and analysis of outcomes and patients’ characteristics–from genetic and biological markers to psychosocial traits–was emphasized. Investigation on immunosuppression protocols is key as immunosuppression-related complications are a major threat to long-term FT recipient survival, often regarded as the trade-off for living a better but shorter life.

### Surgical considerations

A section of the Delphi surveys was dedicated to perspectives on technical and procedural aspects, with a focus on donor and recipient surgeries. Owing to the technical expertise required, this survey section was restricted to the panel subgroup of surgeons and clinicians with a record of accomplishment in the field. FT surgeries differ for the type of aesthetic and functional defects, donor and recipient preparation, and allograft components, which make standardization difficult and oversimplistic. As concerns donor surgery, face allograft procurement entails a complex dissection that requires a significant amount of time, during which donor’s physiology might be jeopardized. To preserve potentially life-saving organs, coordination with solid organ teams becomes of utmost importance.^22^, ^23^, ^24^ According to the panel, initiation of donor surgery with face allograft procurement, and under good hemodynamic conditions, is desirable to preserve facial perfusion and avoid the detrimental effects of high catecholamine load on allograft vasculature.^25^ In case of hemodynamic instability, organ procurement should be given priority, postponing or aborting face allograft procurement. This implies that any procedure should be thoroughly orchestrated based on coordinating algorithms shared by solid organ and vascularized composite allograft teams and based on donor’s physiology.

Prior to face procurement initiation, all face-connected probes (eg, venous access and gastric tubes) should be removed to avoid any interference with allograft procurement and tracheostomy should be performed. Among the factors weighted against organ donation by donor families are concerns about mutilation of the deceased body. In this regard, a respectful return of the donor to the family should be guaranteed according to the family’s wishes (eg, facial mask).

As of today, there is no well-established cutoff for tolerable ischemia time during FT surgery, although it has been shown that prolonged exposure of tissues to ischemic conditions might impact functional recovery and trigger alloimmune activation. According to the convened group, ischemia time should be kept to a minimum to reduce the risk of ischemia reperfusion injury and a cutoff time of 4 hours was advised as desirable.

Recipient surgery should be initiated once face allograft procurement has been completed or considered safely performed by the donor team. Consensus was not reached on the ideal time for initiation of posttransplant rehabilitation. This was likely the result of a restraining statement, which envisaged initiation of rehabilitation soon after the stabilization of patient’s conditions but not before 1 month after surgery.

### Data on patient progress and outcomes

FT teams have shared outcomes of FT as regards functional recovery, aesthetic improvement, revision surgeries, and health-related quality of life.^26^, ^27^, ^28^, ^29^ If we consider FT history, outcome data sets are nonetheless incomplete and heterogeneous in the reported information. Underlying this shortcoming are the small number of procedures performed and lack of data sharing across worldwide institutions. Participants outlined as major barriers issues of data ownership and privacy protection, logistic complexity of data collection, and reliability of completion rate for patient-reported outcome measures. It was also emphasized the need to share negative results, overcoming the competitive culture and willingness to promote a positive perception, favoring transparency. Improvement in data collection and sharing is desirable as it would foster improvement in all areas of policy and practice, from patient and support network assessment to clinical approaches and funding models. In this regard, delegates recommended the employment of formalized processes, ideally of an international data registry for all procedures, to capture data on treatments used and clinical outcomes.

Consensus was reached on the need for combining standardized metrics for outcome evaluation, including quality of life, identity perception, social reintegration, and return to work. FT being a life-improving procedure, patient perspective and experience should be given importance in overall outcome assessment. To overcome the limits posed by accessing patients regularly over a long time, and improve completion rates, participants recommended scheduled electronic administration of questionnaires.

As little is known about patients who are referred to but decide not to proceed with FT, recommendation was given on tracking outcomes for patients enrolled on an FT program, at the time of referral and afterward. This would give insights on the natural history and full range of outcomes of patients living with facial defects and a long-term perspective of benefits and risks of conducting vs not conducting the procedure.

### Definition of success and failure

Lack of definitions of success and failure in FT has been recognized as a critical issue warranting consensus.^30^ Defining success and failure is critical to many areas of FT practice and policy. Among the advantages are the implementation of risks versus benefits analysis, the identification of predictive factors for success and failure, the improvement in pretransplant assessment of candidates and support networks, and the promotion of funding models. Furthermore, from a clinical perspective, defining failure is key to delineation of prevailing conditions underlying unfavorable outcomes. On the other hand, reports of success strengthen FT indication for treatment of severe facial defects and to substantiate it as a standard of care. The panel broadly agreed on the need to establish a consensus definition for FT failure. Several statements were proposed to define “failure” applicability to different clinical scenarios. Following the second Delphi round, agreement was reached on defining failure according to the following criteria:

1. Irreversible deterioration of the face allograft for surgical complications, immunologic rejection, and nonimmune allograft vasculopathy
2. The removal of the face allograft for external conditions (systemic complications, cancer)
3. Death from immunosuppression-related complications
4. Death from “preventable” externalities (eg, suicide, noncompliance, and denial)

In the light of the heterogeneity of the presented scenarios, delegates outlined the need to distinguish among the cohort of failures those related to conditions directly compromising allograft viability from those inherent to patient complications and death.

According to the panel, a satisfactory definition of FT success should frame both the clinical team and patient evaluation, integrating surgical, clinical aspects, and individual reported outcomes.

### Public image and perception

Public perception of FT is significantly affected by the media landscape. Most reports offer narratives centered on procedural aspects, before and after comparisons, and sensational aesthetic outcomes. This representation provides inaccurate views of the procedure and fosters unrealistic expectations, which might be detrimental to patients, support network members, donor families, and funding institutions. The panel expressed a relative consensus on the importance of media narratives in the continued success of FTs, although this aspect was deemed subordinate to issues of greater urgency. According to the panel, FT recipients and network members should be provided with proper instruments to deal with media interests and avoid privacy violation. Less importance was given to clinical team media engagement, as 67% of participants considered communication and public relation teams not necessary. Owing to the heterogeneity and the complexity of media organization within different countries, the development of a standardized practice for media reporting is out of reach. However, a recommendation was given on what should be a useful narrative: patient-centered, reporting individual experience and thoughts, and ideally resulting from long-term views. During the laboratory test, participants also emphasized the importance of attention to language use, particularly in relation to terms such as “deformity,” “abnormality,” and “disfigurement.”

### Financial sustainability

The economic aspects of FT are often overlooked in favor of clinical issues. As the field is evolving, it is likely that the need for financial resources will be a major limitation, in the place of ethical and technical feasibility issues. It is thus imperative to understand how to create financially sustainable FT programs and support patients and their families, guaranteeing equality of access to FT. The estimation of FT costs on a global scale pertains to the complexity of different health care systems. Furthermore, beyond direct costs of transplantation, long-term care, immunosuppression, revision surgeries, and psychological and psychiatric support require significant ongoing resources. According to the panel, financial support, either private or public, is a basic requirement to render FT sustainable. During the laboratory test, participants outlined communication difficulty with funders, when it comes to evaluating FT in terms of cost-benefit analyses. Indeed, the benefits of a life-enhancing procedure are best outlined on a qualitative or even narrative basis, rather than on a quantitative basis. Then, what can be done to motivate funders to reimburse FT procedures? The decision to fund FT should be grounded on the duty of care owed to patients and the actual effectiveness of FT compared to alternative procedures. In this regard, improvement in patient selection criteria, outcome evaluation, and reporting, including quality of life measures, is key to better depict the benefits of transplantation and enable comparative analysis of its cost-effectiveness vs conventional reconstruction. According to most participants, financial resources should not be limited to the procedure itself but should include posttransplant care (long-term follow-up, travel and housing costs, psychological support, and immunosuppressive therapies).

## Conclusions

Collaboration among FT teams on an international basis is crucial for the development of the field of FT. Here we report the results of the first international consensus, which saw the participation of major FT stakeholders, with a wide representation of the teams that had already performed the procedure. The choice of a multidisciplinary panel enabled to capture the perspectives of leading surgeons, clinicians, psychologists and psychiatrists, qualitative researchers, and policymakers on critical issues of FT. The present study was conceived with the idea of laying the groundwork for international cooperation, and establishing a set of recommendations, which should serve as a shared gold standard of care. The discussion among experts also enabled us to outline controversies and areas requiring investigation and further debate. Despite the broad representation of international FT teams, the authors recognize the limit of having had a partial participation of FT centers.

## Funding

The authors received no financial support for the research, authorship, or publication of this manuscript; the research underpinning the Delphi Study and the AboutFace Policy Lab was funded by the UKRI. Grant reference number MR/S017356/1.

## Disclosure

The authors of this manuscript have no conflicts of interest to disclose with regard to the research, authorship, or publication of this article as described by the *American Journal of Transplantation*.

## Data availability

Data sharing is not applicable to this article as all collected and analyzed data are present in the manuscript.

## Declaration of competing interests

The authors declare the following financial interests/personal relationships which may be considered as potential competing interests: Daniel Saleh reports a relationship with Stryker UK Ltd that includes: speaking and lecture fees. Daniel Saleh reports a relationship with Sanofi Genzyme that includes: speaking and lecture fees. Romain Bosc reports a relationship with Alexion Pharma France SAS that includes: consulting or advisory. Romain Bosc reports a relationship with AstraZeneca SAS that includes: consulting or advisory. Sheila Jowsey-Gregoire reports a relationship with US Department of Defense that includes: funding grants. Sheila Jowsey-Gregoire reports a relationship with Mayo Clinic that includes: travel reimbursement.

## Appendix A Supplementary data

Multimedia component1 Supplementary Methods
