# Supplementary material for: International consensus recommendations on face transplantation: A 2-step Delphi study
Source: Am J Transplant. 2024 Jan;24(1):104–14. doi: 10.1016/j.ajt.2023.08.023 (PMC10881406; doi:10.1016/j.ajt.2023.08.023)
Supplement: Multimedia component1 [file mmc1.docx]

Appendix. Delphi Questionnaires – Statements for Delphi 1 and 2

| Definitions | Colour legend |
| --- | --- |
| Consensus: Agreement ≥ 75% (≥ 75% answers “Strongly agree” or “Agree” |  |
| Consensus not reached (No agreement ≥ 75% nor disagreement ≥ 75%) |  |
| Negative consensus: Disagreement ≥ 75% (≥ 75% answers “Strongly disagree” or “disagree” |  |

Sections

1. Patient assessment and selection

2. Face transplant indications

3. Social support networks

4. Clinical framework

5. Surgical considerations

5. Data on patient progress and outcomes

6. Definition of success and failure

7. Public image and perception

8. Financial sustainability

| **Section 1** | | | | |
| --- | --- | --- | --- | --- |
| **Statement** | | **Agreement**  **(%)** | | **Agreed on Delphi 1/2** |
| 1. The development of standardised protocols for candidate assessment and selection for face transplantation is required. | | 97% | | 1 |
| 2. Patient assessment and selection should be handled by multidisciplinary teams of experts, including surgeons, clinicians, psychologists, psychiatrists | | 100% | | 1 |
| 3. During patient selection, individual circumstances must be thoroughly assessed and  considered (e.g. psychological status, personal distress, self-perception) | | 100% | | 1 |
| 4. Patient expectations and perception of “transplant success” should be investigated  and discussed at the time of patient selection and enrolment in a face transplant program | | 100% | | 1 |
| 5. Psychological and psychiatric pre-operative evaluation are mandatory for enrolment  in a face transplant program | | 97% | | 2 |
| 6. The presence of a social support network is a basic requirement for face transplant  candidates. Accordingly, lack of a social support network represents a contraindication to face transplantation. | | 79% | | 2 |
| 7. Psychiatric illnesses are an absolute contraindication to face transplantation | | 47% | | - |
|  | | | | |
| **Section 2** | | | | |
| **Statement** – (*rated by medical experts) | | **Agreement**  **(%)** | | **Agreed on Delphi 1/2** |
| 1. Face transplantation should be considered as a first-line intervention for craniofacial defects involving mid- and central aesthetic units of the face (eyes, nose, upper and lower lips) or so extensive to predict a sub-optimal outcome of conventional reconstruction. | | 100* | | 2 |
| 2. Face transplantation should be considered only when reconstruction of severe craniofacial defects with autologous tissues has been attempted without yielding satisfactory aesthetic and functional outcomes. | | 68* | | **-** |
| 3. Viable facial tissues should be spared at time of transplantation, preferring partial over full face transplantation when feasible, owing to the risk of face allograft failure. | | 70.8* | | - |
| 4. Ballistic/blunt trauma, burns and animal bites causing significant tissue loss to the middle third of the face including nose, lips and eyelids should be considered as indications to face transplantation | | 68* | | - |
| 5. Extensive plexiform neurofibromas or extended defects resulting from cancer excision should be considered as indications to face transplantation.  Combina nello stesso statement patologia benigna (forse più che indicata), e patologia maligna che avevamo scritto addirittura 20 anni disease free. | | 64* | | - |
| 6. Complete loss of the orbicularis oculi and/or orbicularis oris should be considered as indications to face transplantation | | 80* | | 2 |
| 8. A past medical history of benign tumour should not be considered as a contraindication to face transplantation | | 84* | | 2 |
| 9. Extensive facial defects with a past medical history of malign tumour represent a relative indication to face transplantation after a free-disease period of twenty years. | | 56* | | - |
| 10. A past medical history of HIV infection represents an absolute contraindication to face transplantation. | | 32* | | - |
| 11. Donor and recipient gender matching should not be necessarily concordant. | | 55.8 | | - |
| 12. Facial allotransplantation is indicated in paediatric patients with extensive facial defects. | | 29.4 | | - |
|  | | | | |
| **Section 3** | | | | |
| **Statement** | | **Agreement**  **(%)** | | **Agreed on Delphi 1/2** |
| Evaluation of patient social support network should include psychosocial support (positive attitude to promote patient well-being, self-acceptance, social reintegration and return to work) | | 100 | | 1 |
| Evaluation of patient social support network should include practical support (availability to assist the patient on follow-up course, rehabilitation, revision surgeries, return to work and social reintegration) | | 100 | | 1 |
| Evaluation of patient social support network should include financial resources (financial capacity to ensure life-long immunosuppressive therapy, psychological/psychiatric support, rehabilitation, follow-up related costs, travel, and housing costs) | | 85 | | 1 |
| A negative attitude of social support network members may affect the outcome of face transplantation, implying increased risk of patient distress, depression/anxiety and reduced compliance with post-transplant follow-up, and should considered during ft candidate assessment and selection. | | 91 | | 1 |
| Social support network members should be involved as soon as possible in the face transplant process. | | 94 | | 1 |
| Face transplant teams must ensure open discussions on quality of life after transplant, transplant-related complications, allograft loss, chronic immunological rejection with social support members. | | 97 | | 1 |
|  | | | | |
| **Section 4** | | | | |
| **Statement** | | **Agreement**  **(%)** | | **Agreed on Delphi 1/2** |
| Collaboration and sharing of information among clinical teams, on a national and international basis, is necessary to improve single team capacity and ensure the best care to face transplant recipients. | | 94 | | 1 |
| A comprehensive post-transplant follow-up should provide clinical and psychological care to face transplant recipients, handed over multidisciplinary teams of experts | | 100 | | 1 |
| A comprehensive face transplant plan must address salvage contingency strategies to adopt in the unlikely event of face allograft failure/loss | | 100 | | 2 |
| Investigation of predictive factors of success (trauma-related distress, psychological assessment, social outcomes, biomarkers) is required | | 100 | | 1 |
| Investigation and research on immunosuppressive therapy protocols is necessary to minimise the risk of immunosuppression-related complications (e.g. infections, tumours), to which all transplant recipients are inevitably exposed and because of which a shorter than normal life expectancy is expected | | 100 | | 1 |
| Chronic immunological rejection causing late allograft loss is a major challenge in the long-term follow-up of face transplant patients | | 91 | | 1 |
| The development of standardised processes for monitoring immunological rejection of face allografts (follow-up mucosal/skin biopsies, study of vascular changes through echo/MRI, serum antibody titres) is necessary | | 97 | | 2 |
| The risk of chronic immunological rejection should be discussed with face transplant recipients, outlining the possible need of future reconstructions with autologous tissues or a new face transplantation | | 94 | | 1 |
| Facial retransplantation is a valid approach in case of face allograft loss. | | 85 | | 2 |
|  | | | | |
| **Section 5** | | | | |
| **Statement** (*rated by medical experts) | | **Agreement**  **(%)** | | **Agreed on Delphi ½** |
| In the setting of donor surgery, the face harvest should be performed first (before thoracic and abdominal organs are harvested) under good hemodynamic donor conditions and to avoid the use of massive levels of catecholamines that could have detrimental effects on the allograft vasculature. | | 88* | | 2 |
| In case of donor hemodynamic instability, organs procurement must have the priority and face allograft harvest should be aborted or postponed. | | 92* | | 2 |
| Face donor should be free of any kind of probes (i.e. venous access, nasogastric tube) so to avoid any interference with the allograft procurement. | | 76* | | 2 |
| Tracheostomy is mandatory to perform face allograft procurement. | | 80* | | 2 |
| A resin mask should be produced to allow a respectful return of the donor to his/her family. | | 92* | | 2 |
| Recipient surgery should be started once face allograft procurement has been done or has been considered safely performed by the donor team. | | 76* | | 2 |
| Ideal ischemia time of face allograft before revascularization should not exceed more than four hours. | | 92* | | 2 |
| Rehabilitation of face transplant recipients should start as soon as possible but not before 1 month after surgery and when general conditions are stable. | | 60* | | - |
|  | | | | |
| **Section 6** | | | | |
| **Statement** | | **Agreement**  **(%)** | | **Agreed on Delphi ½** |
| Formalised processes (e.g. compulsory registry) are required to ensure timely reporting and sharing of post-transplant outcomes and events (e.g. rejection episodes, post-transplant complications, motor and sensory recovery, post-transplant quality of life) among face transplant teams, on a national and international basis. | | 97 | | 2 |
| Data collection and sharing is necessary to improve patient and social support network evaluation | | 97 | | 1 |
| Data collection and outcomes analysis are necessary to promote the development of funding models for face transplantation | | 97 | | 1 |
| Standardised metrics are required to evaluate post-transplant individual outcomes (quality of life, self-perception, social reintegration, return to work) | | 97 | | 1 |
| Electronic scheduled collection of questionnaires on patient’s perception and experience following transplantation (PROMS, PREMS, QoLs) may improve completion rates | | 91 | | 2 |
| The quality of life and outcomes of patients receiving face transplantation should be compared with that of those referred for face transplantation, who decide not to proceed with the surgery, with the aim of understanding potential benefits and the impact of face transplantation on patients with severe craniofacial defects. | | 94 | | 1 |
|  | | | | |
| **Section 7** | | | | |
| **Statement** | | **Agreement**  **(%)** | | **Agreed on Delphi ½** |
| A consensus definition of failure in the setting of face transplantation is necessary | | 88 | | 1 |
| Reaching a consensus definition of failure in the setting of face transplantation is required to improve patient selection and foster investigation on pathophysiological mechanisms of failure-related causes | | 91 | | 1 |
| A comprehensive definition of failure in the setting of face transplantation must include all conditions leading to irreversible deterioration of the face allograft (e.g. surgical complications, immunological rejection, allograft vasculopathy) | | 97 | | 2 |
| A comprehensive definition of failure in the setting of face transplantation must include all external conditions prompting removal of the face allograft (e.g. infections, cancer, severe systemic complications) | | 97 | | 2 |
| A comprehensive definition of failure in the setting of face transplantation must also include “preventable” externalities related to patients’ compliance and post-transplant experience (e.g. suicide, non-compliance to immunosuppressive therapy and follow-up) | | 97 | | 2 |
| In my opinion a comprehensive definition of failure in the setting of face transplantation must include patient death for transplant and immunosuppressive therapy-related complications (tumour occurrence, opportunistic infections) | | 88 | | 2 |
| In my opinion it would be advisable to distinguish failures related to conditions directly affecting viability of face allografts from deaths related to suicide, non-compliance, and immunosuppressive-related complications | | 82 | | 2 |
| Being a life-enhancing procedure, success in the setting of face transplantation must be evaluated beyond clinical and surgical aspects, considering patient’s post-transplant self-perception, experience, and quality-of-life change | | 100 | | 1 |
| Success in the setting of face transplantation is strongly affected by patient and social support network pre-transplant expectations on outcomes and post-transplant course. Accordingly, any effort should be done to depict to face transplant candidates and their support networks practical aspects of life after transplantation (rehabilitation, need of revision surgeries, psychological support, immunosuppressive therapy, follow-up visits) | | 97 | | 1 |
|  | | | | |
| **Section 8** | | | | |
| **Statement** | | **Agreement**  **(%)** | | **Agreed on Delphi ½** |
| Comprehensive, long-term, patient-centred narratives, including individual experience, the role of social support network and challenges faced after face transplantation should be encouraged over brief reports and before-and-after comparisons. | | 82 | | 1 |
| Face transplant programs should provide patients with proper instruments to deal with media interest and to avoid violation of their privacy | | 97 | | 1 |
| Clinical teams should be supported by dedicated communication and public relations teams to understand benefits and risks of media engagement | | 21 | | **-** |
|  | | | | |
| **Section 9** | | | | |
| **Statement** | **Agreement**  **(%)** | | **Agreed on Delphi ½** | |
| I recognize the presence of inequality of access to face transplantation based on economic issues in the country where I work | 29.4 | | **-** | |
| Funding systems are necessary to ensure face transplant economic sustainability for patients and to avoid the structural bias of face transplantation access | 82 | | 1 | |
| Data on post-transplant outcomes and QoL measures may be an instrument to depict the benefits of face transplantation as opposed to other treatments or non-treatment to funders | 94 | | 1 | |
| Standardization of patient assessment and selection may provide an additional tool to encourage funding of face transplantation | 97 | | 1 | |
| Institutions should provide the necessary long-term financial support to face transplant recipients as a gold-standard of care. This must include costs of housing, travel, follow-up, biopsies, immunosuppressive therapy. | 73 | | - | |
